# An Audit of Operating Room Time Utilization in a Teaching Hospital: Is There a Place for Improvement?

**DOI:** 10.1155/2014/431740

**Published:** 2014-03-13

**Authors:** George Stavrou, Stavros Panidis, John Tsouskas, Georgia Tsaousi, Katerina Kotzampassi

**Affiliations:** ^1^Department of Surgery, Faculty of Medicine, Aristotle University of Thessaloniki, 54636 Thessaloniki, Greece; ^2^Department of Anesthesiology, Faculty of Medicine, Aristotle University of Thessaloniki, 54636 Thessaloniki, Greece

## Abstract

*Aim*. To perform a thorough and step-by-step assessment of operating room (OR) time utilization, with a view to assess the efficacy of our practice and to identify areas of further improvement. *Materials and Methods*. We retrospectively analyzed the most ordinary general surgery procedures, in terms of five intervals of OR time utilization: anaesthesia induction, surgery preparation, duration of operation, recovery from anaesthesia, and transfer to postanaesthesia care unit (PACU) or intensive care unit (ICU). According to their surgical impact, the procedures were defined as minor, moderate, and major. *Results*. A total of 548 operations were analyzed. The mean (SD) time in minutes for anaesthesia induction was 19 (9), for surgery preparation 13 (8), for surgery 115 (64), for recovery from anaesthesia 12 (8), and for transfer to PACU/ICU 12 (9). The time spent in each step presented an ascending escalation pattern proportional to the surgical impact (*P* = 0.000), which was less pronounced in the transfer to PACU/ICU (*P* = 0.006). *Conclusions*. Albeit, our study was conducted in a teaching hospital, the recorded time estimates ranged within acceptable limits. Efficient OR time usage and outliers elimination could be accomplished by a better organized transfer personnel service, greater availability of anaesthesia providers, and interdisciplinary collaboration.

## 1. Introduction

Operating theatres' cost constitutes a huge investment of healthcare resources, approximating one-third of total hospital budget [1, 2]. Thus, there is an increasing interest in providing an “efficient” anaesthetic and surgical service [3], to make operations the largest potential source of income [1, 2]. However, case cancellations on the day of surgery, due to suboptimal utilization of theatre time [4–7], is a well-recognized problem in hospitals, ranging from 10% to 40% across different health care systems worldwide, 60% of which could potentially be avoided [8–10].

Delays and consequent cancellations of surgical procedures are arguably an issue of health care quality [8] as well as a major cause of waste of health resources [2, 10]. As a consequence, they prolong the duration of hospitalization causing anxiety, frustration, anger, emotional involvement [11], and inconvenience to patients and their families [12], quite apart from increasing the cost in terms of working days lost and disruption to daily life. The most common causes of cancellation are the patient being unfit for surgery and suboptimal utilization of theatre time [6, 9], with the latter leading to case delays. Several studies have shown organizational issues to be the cause, such as a lack of sufficient theatre time, overrunning of lists, and poor coordination between staff [6, 10, 13, 14].

Despite the growing interest in utilization of hospital health care resources, only one study so far involving gynecological population has looked into each step of a patient's journey through the operating theatre, in order to elucidate the factors which make this passage inefficient and lead to poor operating theatre utilization [15]. Thus, the purpose of the present study was to perform a thorough and step-by-step assessment of the utilization of operating theatre time by validating the time estimates of five typical stages of perioperative time management, in relation to some of the most ordinary operations in general surgery, with a view to assess the efficacy of our practice and to identify areas of further improvement.

## 2. Materials and Methods

This study was conducted after the approval of Scientific Committee for Medical Research Ethics. During a 12-month period, we retrospectively analyzed the operating room (OR) database to retrieve only the cases involving six predetermined types of general surgery procedures, classified according to their surgical impact as minor (inguinal hernia), moderate (postop ventral incision hernia and laparoscopic cholecystectomy), and major (total gastrectomy, total colectomy, and abdominoperineal colonic resection). These types of operations were selected on the basis of variability of duration or severity and the degree of patients' vulnerability to perioperative complications.

The inclusion criteria involved the exclusive fit to one of the six operations included in the study protocol, elective operations, and general anaesthesia with endotracheal intubation cases. The combination with epidural/spinal or nerve block was grounds for exclusion.

For each individual, five different time intervals were collected from the OR chart: induction to anaesthesia, preparation for surgery, duration of surgical procedure, recovery from anaesthesia, and transfer from OR to the postanaesthesia care unit (PACU) or intensive care unit (ICU). The time period referred to as “induction to anaesthesia” (T1) includes preparation of each patient by a qualified anaesthesia nurse, catheterization of peripheral or central vessels (veins and arteries), and finally tracheal intubation, as appropriate. The time period referred to as “preparation for surgery” (T2) includes proper positioning of the patient, insertion of urinary bladder catheter, possibly the need for adjustment of accessories to the operating table, patient disinfection, and draping and surgeon disinfection gowning and gloving. “Duration of operation” (T3) is exclusively the time period between the initial cutting and the putting of a drape over the final stitches. The time period referred to as “recovery from anaesthesia” (T4) includes, for the minor and medium severity operations, the time from the last stitch to the patient extubation and adequate recovery from anaesthesia to the point of transfer to the PACU, while for major operations it includes the time from the last stitch to the time either the patient was extubated and had slightly recovered in OR, or for clinical reasons, it has been decided in advance to be transferred intubated to the ICU. Finally, the time period referred to as “patient taken from theatre” (T5) represents the time needed for the porter to come and to transfer the patient out of the OR towards the PACU or ICU.

### 2.1. Statistical Analysis

One-way analysis of variance (ANOVA) with Bonferroni correction was conducted to compare means of continuous variables and normal distributed data, while a nonparametric rank test, the Kruskal-Wallis test, was used to compare means in the case of nonnormally and noncontinuously distributed data. Normality of data was assessed by Kolmogorov-Smirnov test. Subgroup comparisons of categorical variables were assessed by a chi-square test. For all statistical procedures, a *P* value of less than 0.05 was considered statistically significant. Data were analyzed using SPSS version 18.0 (SPSS Inc., Chicago, IL, USA).

## 3. Results

Data from a total of 548 patients were subtracted from the OR database anonymously.

According to the surgical impact classification described, 6.8% (*n* = 37) of the patients were found to have undergone minor surgical procedures, 68.2% (*n* = 374) medium surgical procedures, and 25% (*n* = 137) major surgical procedures.

Detailed data regarding the total time that the patient remained in the OR in the different group of operations are presented in Table 1. With the exception of duration of surgery, the remaining time intervals in each study group were comparable. Only in major surgical impact cases the time spent for induction to anaesthesia presented a subtle augmentation regarding recovery from anesthesia and transfer to PACU or ICU (*P* = 0.034 and  *P* = 0.018, resp.).

**Figure 1:**
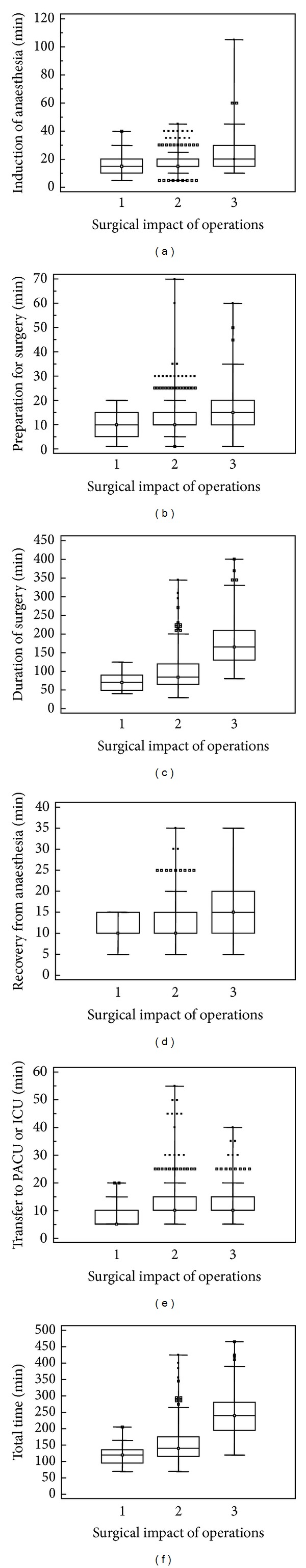
Time intervals and total time according to the impact of the surgical procedure. Box and whisker plots of each setup according to the operations' surgical impact. Data are expressed in minutes as median (range); 1: minor surgical impact; 2: medium surgical impact; 3: major surgical impact. *P < 0.05, **P < 0.01,***P < 0.001 indicate statistical significance of each setup versus minor surgical impact procedures.

Data obtained from the classification of the studied time intervals according to the surgical impact of the involved operations is presented in Figure 1. In the time spent for anaesthesia procedure there is a statistically significant difference (*P* = 0.000) among the mean time spent for general anaesthesia preparation in minor or even medium surgical impact operations (16-17 min) compared to major procedures (25 min). In this way we could empirically divide the time for anaesthesia into that for the basic needs (peripheral vein insertion plus anaesthesia induction and endotracheal intubation) and that used for applying extra catheterization (central venous and arterial lines), estimated to be approximately 15 to 25 min, respectively. In the same manner, there is a statistically significant difference between time used for patients to be subjected to minor and medium operations compared to major surgery. In regard to the duration of operation, there is also a statistically significant difference (*P* = 0.000) between minor and medium operations, as well as between medium and major surgery, both assessed either individually or as groups.

Concerning the time for anaesthesia recovery, the studied groups also differed significantly (*P* = 0.000). It is noteworthy that 2.4% (*n* = 9) of the patients in medium and 32.8% (*n* = 45) in major surgery were transferred intubated in ICU, while all patients in minor operations were extubated in OR (*P* = 0.000).

Finally, the time spent waiting for the transport personnel to transfer the patient out of OR presented a statistically significant difference between minor surgery patients in relation to both medium and major surgery (*P* = 0.006).

## 4. Discussion

Our data showed that in our teaching university hospital the time intervals of five typical stages of OR time utilization concerning the most ordinary elective operations in general surgery ranged within acceptable limits and were comparable to the time estimates of published operating lists. With the exception of transportation to PACU or ICU where the most outliers were recorded, excessive delays were not our case.

Delays of surgical procedures as well as cancellations due to overrunning of the lists and suboptimal utilization of theatre time are common occurrences throughout the world. There have been many reports to show that this results in wastage of OR time and prolongation of patients' hospitalization, leading to increased cost and emotional involvement [12]. On the other hand, several reports aimed at identifying the causes of delays and constructing sophisticated models or simple algorithms for more accurate prediction of time needed for each individual intervention, to avoid overbooking and optimize the efficient use of OR time [2, 3, 16]. However, many unpredictable factors have been found to interfere, leading finally to operation delay, despite the operation time itself being well estimated. In the present study we tried to analyze, in a step-by-step procedure, the time spent in surgical procedures with escalating surgical impact and, in order to keep a degree of similarity, we used only elective operations and only those performed under general anaesthesia, including tracheal intubation. Therefore, we ensured that the time periods of anaesthesia and of operation are proportionally similar, enabling us to scrutinize the remaining three time periods (preparation for surgery, recovery from anaesthesia, and transportation out of OR) for more obscure causes of delay. We deliberately omit a parameter studied by many others which is patients delay in entering the OR. This was decided for two reasons: first because there is a standard practice the patient being transferred from the ward to wait in the OR corridor, and second because the required time for transfer personnel to leave the OR, go to the ward, and bring back the patient is not the same in the morning as for later cases (unpublished data).

In an effort to estimate the time needed for induction of anaesthesia, Koenig et al. [17] recorded a mean time of about 10 min needed for applying only a laryngeal mask, about 15 min for tracheal intubation only, and about 25 min for intubation plus arterial line; the differences between the anaesthetic techniques regarding induction time are statistically significant, which are in line with our findings. In the same manner, in an audit of females subjected to gynaecology surgery, the time interval for induction of general anaesthesia ranged between 5 and 17 min in cases when anaesthesia was provided via only a peripheral vein [15]. Complex techniques involving nerve blocks or the placement of a central venous or an epidural catheter are likely to be more time consuming, which is why we excluded such cases from the study protocol. Moreover, teaching or patient case mix might also influence the induction time [18], as does the individual anaesthetist's performance level. Even for experienced anaesthetists, it is often difficult to predict the duration of anaesthesia induction for a specific case [19]. We should underline the fact that the time needed for induction to anesthesia was proportionally longer for simple compared to less simple procedures, which might be attributed to the involvement of younger, less experienced trainees in simple cases versus more skillful trainees for major surgery. Additionally, in several cases, central venous or radial artery catheter insertion was carried out simultaneously following tracheal intubation, thus ensuring some minutes being saved over the total operation time. In any case, process time might be influenced by the performance level of the individual anaesthetist, or by other specific factors such as shortage of instruments or of staff [17].

Concerning time for surgery preparation, Mazzei [20] reported that surgical incision in an academic hospital was performed 21 to 49 min after the patient was brought into the OR. This time interval is comprised of the time for induction to anaesthesia (8 to 32 min) and the surgical preparation time required for the various surgical procedures, which is estimated to be about 20 min. Delays in that time period could be mainly attributed to system deficiencies [3, 10], such as technical errors or equipment inefficiency, contamination of instruments or drapes required, failure in communication between members of the operating team, or defects in the staff performance [21, 22]. It is of interest to comment that, in our material, the time needed for major surgery preparation was statistically longer, not proportionally analogous to the preparation needed for minor surgery. In other words, while it might be well accepted that the time needed for patient preparation for abdominoperineal colonic resection should be more than double compared to the time for inguinal hernia repair, this is not our case. This finding could be attributed both to nurses who, by thinking “it is an easy case; let us relax a little,” delay preparation of the OR and the patient and to assistants who lose time in a similar way. These slightly odd findings have led us to suggest the existence of differences between the personnel involved, for instance, more capable and qualified nurses in major operations.

Saha et al. [15] in gynecological procedures recorded a median interval of 148 min from transportation to OR preoperatively up to PACU postoperatively, the performance of the surgery itself having taken up the largest slice of this time interval (median 81 min). Broadly speaking, teaching and university hospitals in general were found to have much higher cancellation rates, and the intervention of residents should be one of the main causes of OR time prolongation and thus case cancellation [10]. It is of interest to note the fact that the presence or participation of a resident physician was found to prolong the duration of surgery by up to 70%, with accordingly increased costs [23, 24], while the teaching of an anaesthesia resident seems to delay the anaesthesia procedure by 2-3 min [24, 25]. Our centre is a teaching hospital and the duration of operations might be influenced by the need to train, as the time of anaesthesia induction do. However, the performance time for each individual operation does not exceed the time reported by others [6], although this is not the topic of the present research.

Time for “recovery from anaesthesia” is a little obscure, since in the group of major interventions the complicated cases were moved to the ICU for extubation; thus the time spent in OR was shorter, and consequently the difference between them and those of minor surgery appeared not to be as significant as it was expected. In a similar clinical setting and in accordance with our findings for minor and medium surgical impact operations, the reported wake-up times ranged between 5 and 11 min. However, in cases undergoing major surgery, the time for recovery from anesthesia was relatively prolonged, as the emersion of clinical implications necessitates the provision of a smoother and better-controlled reverse from anaesthesia. Nevertheless, by leaving the patient to take his time for recovery, the available time for the following operation is considerably deteriorated, a situation which, in certain situations, entails possible cancellation of the remaining cases of the surgical list of the day. To deal with this issue several investigators suggested proper titration of anesthetic agents, guided by monitoring of depth of hypnosis, to thus minimize anesthesia-controlled time, as well as to apply a laryngeal airway mask instead of an endotracheal tube, as appropriate [24, 26]. For further promoting the effective usage of OR time, Saha et al. [15] proposed two anaesthetists to be available at the end of surgery; one for the anaesthesia reverse and the other for the next anaesthesia induction, as well as a recovery area being available with sufficient capacity to accept postoperative patients without delay. Nevertheless, existing data report that, by providing an extra anaesthetist and anaesthetic nurse, the midlist gap times are successfully reduced by 20%, but adding an extra case overruns the time by 118% more than greater gaps did [27].

Finally, transporting the patient from OR to the PACU or ICU takes about the same time as for recovery from anaesthesia, the shorter time attributed to minor surgical impact procedures. This may be partially explained by the fact that, in order to avoid waiting for the porter especially if the case is the last on the list, the assistants/residents move the patient by themselves. We can, here, underline the lack of transfer personnel, who may, sometimes, take up to half an hour to arrive, leading to a considerable OR time waste between operations. Saha et al. [15] extrapolate their results to a typical 4 h operating theatre session consisting of three to four procedures, reporting up to 60 min of the surgeons' time being lost while waiting between cases. Their results are to some extent consistent with the study by Hopkins et al. [28] involving patients undergoing ear-nose-throat surgery, concluding that almost one-third of the delay during surgical lists can be attributed to improper organized portering service. Furthermore, Wong et al. [22] estimated that the cost of nursing and OR attendants for each 10-minute delay approximated $18, based on the current hourly pay rate for both, and for a mean rate of 135 delays per year this was translated to $2430 annually for a single OR. By assuming that the other ORs had a similar prevalence of delays, they extrapolated the cost to the entire surgical department, calculating a total cost because of delays of about $138 857 per year.

Several limitations could be acknowledged in the present study. Firstly, our study population represents a specific study group limited to those undergoing elective general surgery procedures operated in a large teaching hospital. Albeit this enhances the reliability, it weakens the generalizability of our findings. Secondly, an established classification of the system delays is still lacking, which limits the comparable validation of our findings to those incorporated from similar clinical settings.

## 5. Conclusion

Albeit our study was performed in an teaching university hospital for both surgeons and anaesthetists, as well as for nurses, the time intervals of five typical stages of OR time utilization concerning the most ordinary elective operations in general surgery ranged within acceptable limits, being comparable to the time estimates of published operating lists. Special emphasis towards improvement of OR time efficiency and outliers elimination should be paid on a sound organizational structure of transfer personnel service in conjunction to the augmented availability of anaesthesia providers and interdisciplinary collaboration.

## Figures and Tables

**Table 1:** Total time and time intervals according to the impact of the surgical procedure.

Operation	Total time	Time points				
		T1	T2	T3	T4	T5
Minor	118 (29)	16 (7)	10 (4)	72 (24)	11 (5)	8 (4)
Moderate	150 (51)	17 (7)	13 (7)	96 (47)	12 (9)	12 (10)
Major	245 (69)	25 (12)	16 (9)	180 (67)	15 (6)	14 (8)
Sum (all types)	172 (67)	19 (9)	13 (8)	115 (64)	12 (8)	12 (9)

Data are expressed in minutes as mean (SD).

T1: induction to anaesthesia; T2: preparation for surgery; T3: duration of surgery; T4: recovery from anaesthesia; T5: transfer from OR to PACU or ICU.
